# Gedunin modulates cellular growth and apoptosis in glioblastoma cell lines

**DOI:** 10.1002/cnr2.2051

**Published:** 2024-05-04

**Authors:** Michael Stouffer, Elizabeth Wandling, Lindsay Dickson, Stacy Lin, Huanyun Duan, Erika Powe, Denise Jean‐Louis, Amit K. Tiwari, Samson Amos

**Affiliations:** ^1^ Department of Pharmaceutical Sciences Cedarville University School of Pharmacy, Cedarville University Cedarville Ohio USA; ^2^ Department of Pharmaceutical Sciences College of Pharmacy, University of Arkansas for Medical Sciences Little Rock Arkansas USA

**Keywords:** apoptosis, cell proliferation, gedunin, glioblastoma cell lines, signaling

## Abstract

**Background:**

Glioblastomas are characterized by aggressive behavior. Surgery, radiotherapy, and alkylating agents, including temozolomide are the most common treatment options for glioblastoma. Often, conventional therapies fail to treat these tumors since they develop drug resistance. There is a need for newer agents to combat this deadly tumor. Natural products such as gedunin have shown efficacy in several human diseases. A comprehensive study of gedunin, an heat shock protein (HSP)90 inhibitor, has not been thoroughly investigated in glioblastoma cell lines with different genetic modifications.

**Aims:**

A key objective of this study was to determine how gedunin affects the biological and signaling mechanisms in glioblastoma cells, and to determine how those mechanisms affect the proliferation and apoptosis of glioblastoma cells.

**Methods:**

The viability potentials of gedunin were tested using MTT, cell counts, and wound healing assays. Gedunin's effects on glioma cells were further validated using LDH and colony formation assays. In addition, we investigated the survival and apoptotic molecular signaling targets perturbed by gedunin using Western blot analysis and flow cytometry.

**Results:**

Our results show that there was a reduction in cell viability and inhibition of wound healing in the cells tested. Western blot analysis of the gene expression data revealed genes such as EGFR and mTOR/Akt/NF kappa B to be associated with gedunin sensitivity. Gedunin treatment induced apoptosis by cleaving poly ADP‐ribose polymerase, activating caspases, and downregulating BCL‐xL. Based on these results, gedunin suppressed cell growth and HSP client proteins, resulting in apoptosis in glioblastoma cell lines.

**Conclusion:**

Our data provide in vitro support for the anticancer activity of gedunin in glioma cells by downregulating cancer survival proteins.

## 1 INTRODUCTION

Glioblastomas (GBM) are aggressive brain tumors with uncontrolled, fast‐growing cells that invade normal brain parenchyma. In the United States of America, epidemiological studies indicate that there is an increase in the incidence of glioblastomas annually.^1^ The treatment of glioblastoma remains challenging. Chemotherapy with temozolomide with radiation and surgical intervention is the main treatment for glioblastomas.^2^ Even with this multimodal treatment approach, glioblastomas have a poor prognosis and patient quality of life and a survival rate of 12–15 months.^3^ One of the key limitations to the development of therapeutic agents to treat glioblastoma is obtaining molecules with good central nervous system (CNS) penetrability. There are reports in the literature on strategies such as using nanoparticles to circumvent this limitation.^4^

Glioblastoma initiation, progression, and development depend heavily on genetic events, alterations, and signaling pathways. The epidermal growth factor (EGF) receptor is amplified and overexpressed in gliomas.^5^, ^6^ EGFR vIII amplification occurs in about 20%–50% of glioblastomas.^7^ Heat shock proteins (HSPs) are elevated in glioblastomas.^8^, ^9^ Several HSPs, such as HSP27 and HSP90, are expressed at an increasingly high level in glioblastomas due to genetic and metabolic changes. The HSP90 protein is particularly relevant to the study. The abnormal changes result in rapid growth and treatment resistance.^10^ HSP90 functions as a molecular chaperone by regulating the conformational stability of several client proteins such as EGFR, Akt, Raf, MEK, focal adhesion kinase (FAK), PDGFR, Cdk‐4, ‐6, ‐9.^11^ In malignant gliomas, some of these proteins are overexpressed or mutated. In several oncological models, the suppression of the Hsp90 has been shown to cause signaling protein degradation involved in cellular growth and proliferation, regulation of cell cycle checkpoints, and induction of programmed cell death.^12^ HSP90 client proteins such as the EGFR‐PI3K‐AKT‐mTOR signaling play a critical role in several cellular processes, including cell proliferation, apoptosis, cell growth and differentiation, and cell metabolic pathways, contributing to tumor survival and progression.^13^ Additionally, Hsp90 interacts with the Akt protein to prevent proteasomal degradation.^14^ This further sustains the PI3K/Akt signaling and promotes cancer cell survival. Subramani et al. showed that gedunin decreased the proliferation of pancreatic cancer cells by attenuating the phosphorylation of PI3K, AKT, and mTOR.^15^ Therefore, HSP90 may be a potential therapeutic target, and inhibiting this molecular chaperone may aid in reducing invasive glioblastoma growth.

**FIGURE 1:**
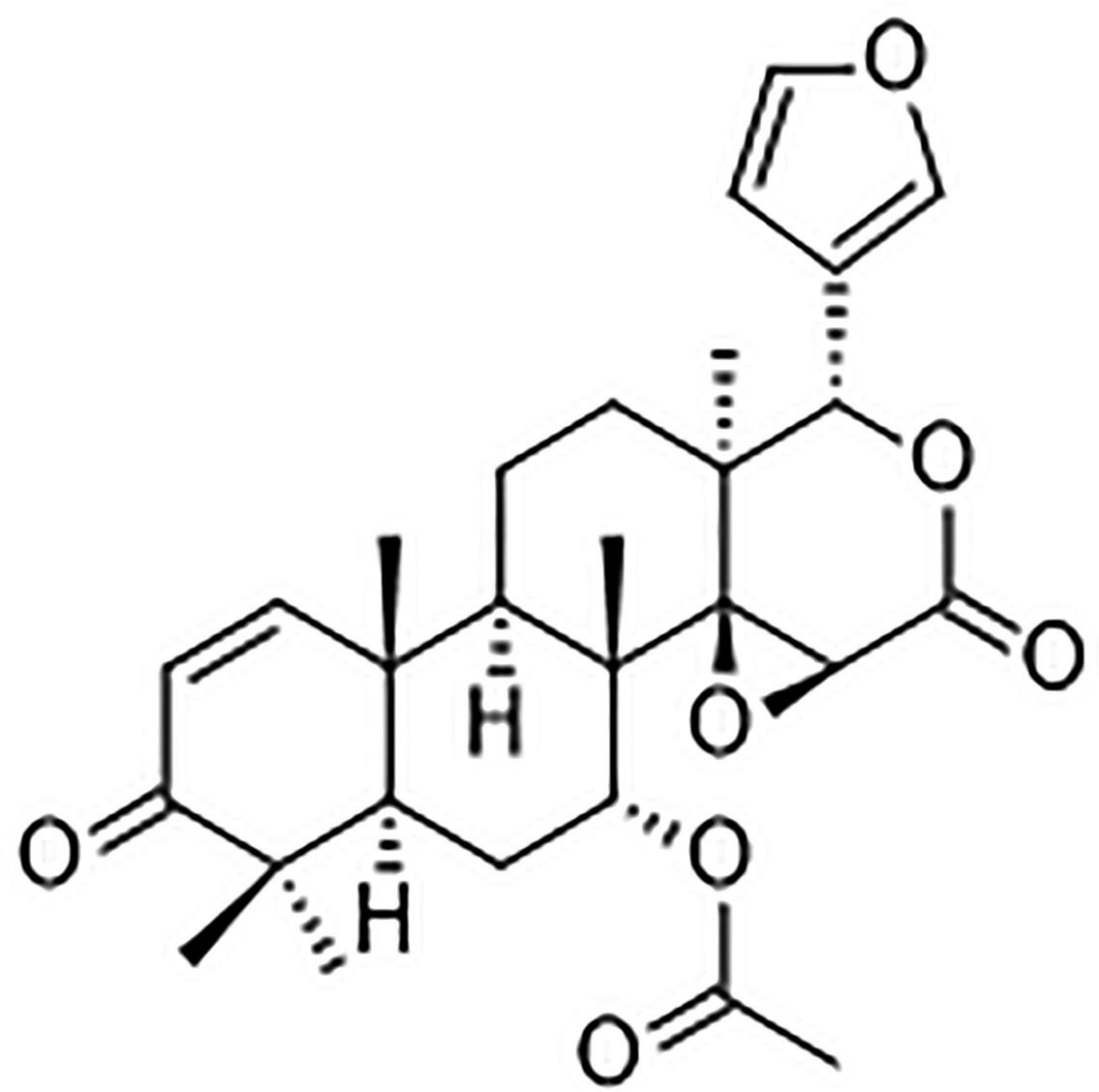
Structure of gedunin.

Various natural products can act as therapeutic agents, and compounds such as alkaloids, flavonoids, polyphenols, and terpenoids have been proven effective in treating various diseases, including cancer.^16^, ^17^ Gedunin is a pentacyclic triterpene with clinical potential as an HSP90 inhibitor (Figure 1). Gedunin was isolated from neem, *Azadirachta indica*.^18^, ^19^ Although traditionally used to treat malaria, gedunin has shown some anticancer properties.^20^ Evidence suggests that gedunin retards the growth and proliferation of many cancer cell lines by disrupting HSP90 folding and causing the breakdown of HSP90‐associated proteins.^15^, ^21^, ^22^ Gedunin has been shown to inhibit secreted and some cytoskeletal proteins resulting in decreased invasive phenotypes.^23^ A major consideration in the management of glioblastoma is the ability of the drug to cross the blood–brain barrier. Gedunin, even though effective in vitro, presents a challenging pharmacokinetic profile. An initial in vivo study using Sprague–Dawley rats with labeled gedunin indicated that it peaked by the eighth hour via an oral route. The radiolabeled drug was also seen in urine at 10 h and in feces at 24 h. Data obtained indicated that the labeled gedunin decreased to background levels in 4 days. Based on this exploratory study of the absorption, distribution, metabolism, and excretion, gedunin is poorly absorbed orally and rapidly cleared in the system.^24^, ^25^

Our laboratory investigated the molecular mechanisms of gedunin's effects on glioblastoma cells. To determine the molecular signaling pathways modulated in GBM cells following gedunin treatment, we examined the effects of gedunin on the growth profile of GBM cell lines with different genetic alterations.

## 2 MATERIALS AND METHODS

### 2.1 Cell lines and reagents

We procured human glioblastoma cell line U87 from the American Type Culture Collection (ATCC, VA, USA), and U251 and U87 EGFR vIII cells were obtained from Dr. Isa Hussaini's Lab, University of Virginia, Charlottesville, VA. We obtained U251 EGFR and U251 EGFR vIII from Dr. James Mandell's University of Virginia, Charlottesville, VA. All glioma cells were grown in DMEM or MEM‐alpha, to which we added 10% fetal bovine serum (FBS) and 1% penicillin/streptomycin (Sigma Millipore, St. Louis, MO). The cells were cultured and placed in an incubator at 37°C in a humidified 5% CO_2_.

### 2.2 Reagents

Gedunin (CAS no. 2753‐30‐2, Cat no. 3387/2 with >98.9% purity) procured from R & D Systems (Minneapolis, MN) was dissolved in DMSO and sterile filtered through a 0.22‐μm filter. MTT (3‐(4, 5‐dimethylthiazol‐2yl)‐2,5‐diphenyltetrazolium bromide) and lactate dehydrogenase (LDH) assay kits were sourced from Promega (Madison, WI). Antibodies were sourced from Cell Signaling Technologies (Danvers, MA). Antibodies directed against EGFR, (#4267 1:1000 dilution), Phospho‐EGF Receptor (Tyr1068) Antibody (#2234, dilution 1:1000), Phospho‐Akt (Ser473) (#4060, dilution 1:1000), Phospho‐mTOR (Ser2448) Antibody (#2971, dilution 1:1000), Phospho‐NF‐κB p65 (Ser536) (93H1) Rabbit mAb (#3033 dilution 1:1000), NF‐κB p65 (D14E12) Rabbit Ab (#8242 dilution 1:1000), Phospho‐p70 S6 Kinase (Thr421/Ser424) Antibody (#9204, dilution 1:1000), EGF Receptor (D38B1) Rabbit mAb (#4267 dilution 1:1000), poly ADP‐ribose polymerase (PARP) Antibody (#9542, dilution 1:1000), Cleaved Caspase‐3 (Asp175) Antibody (#9661 dilution 1: 1000), Bcl‐xL (54H6) Rabbit mAb (#2764 dilution 1:1000), Anti‐Bad Antibody (C‐7): sc‐8044 dilution 1:1000), Anti‐α‐Tubulin antibody, Mouse monoclonal (Sigma‐Millipore (St. Louis, MO. T6199, dilution 1:5000) were used in the Western blot analyses.

### 2.3 Cell count using trypan blue exclusion

Glioma cell lines (1 × 10^5^ cells/well) were cultured in 6‐well plates. The cell lines were treated with gedunin (0–20 μM) for 48 h at 37°C. After 48 h, the cells were dissociated using trypsin, 0.25%. The trypsinized cells were diluted with media and centrifuged (120 × g, 5 min). The media and trypsin were decanted, and then 5 mL of media was added to the cell pellet. We added trypan blue (100 μL) to the cell suspension (100 μL), mixed it, and counted using CytoSmart (Hillsborough, NJ). The results were plotted on dot plot graphs.^26^

### 2.4 Cell viability assay

The antiproliferative and viability effects of gedunin were assessed using the MTT assay. Briefly, U251, U251 EGFR, U251 EGFR vIII, U87, and U87 EGFR vIII cells were cultured in 96‐well plates at 1 × 10^5^ cells per well. After 24 h, gedunin (0–20 μM) was added to the cells for 48 and 72 h. Then, we added 10 μL of the MTT dye and incubated it for 4 h at 37°C. The different sets of experiments were stopped by adding the stop solution. The color absorbance was read at a wavelength of 560 nm on a spectrophotometer (Promega Glomax®, Madison, WI).

### 2.5 Cell apoptosis assay

U251 glioma cell line was treated with gedunin (5–20 μM). After 72 h treatment, the cells were trypsinized and centrifuged. The cell pellet was subjected to Annexin V/FITC stain according to standard protocol. The rate of apoptosis of the stained cells was determined using a BD Accuri C6 Plus software.^27^ The data analysis was done as described by Yu et al.,^28^

### 2.6 Western blot analysis

The key cellular signaling proteins perturbed following treatment with gedunin were examined by Western blots according to the methods of Li et al.^29^ Briefly, glioma cell lines were cultured and exposed to gedunin (0–20 μM). The cell protein was extracted using radioimmunoprecipitation assay lysis buffer from Cell Signaling Technologies. Protein extract quantification was determined using the bicinchoninic acid assay. Equal amounts of proteins (20 μg) were separated on a 10% SDS‐polyacrylamide gel, transferred onto PVDF transfer membranes (Millipore, St. Louis, MO), blocked for 1 h, and treated with either monoclonal or polyclonal primary antibodies. After 16 h at 4°C, the membranes were washed with PBST followed by incubation with horseradish peroxidase‐conjugated secondary antibodies. Protein detection was done using enhanced chemiluminescence with Western Blot Imager Azure 300 Q (Azure Biosystems Dublin, CA).

### 2.7 Colony formation assay

Colony formation in gedunin‐treated U251 and U251 EGFR cells was determined as described by Hu et al.^30^ and Zhao et al.^31^ Briefly, GBM cell lines U251 and U251 EGFR were seeded at a density of 500 per well in 6‐well plates and treated with for 24 h in serum‐free media. The media was changed every 3 days with media containing FBS, followed by incubation at 37°C in 5% CO_2_ with continued culturing for 2 weeks (14 days) to allow colony formation. Following this, the cells were washed with PBS and fixed with 4% paraformaldehyde. After staining with 0.5% crystal violet, the numbers of colonies were counted. All experiments were carried out in triplicate.

### 2.8 Wound healing (scratch) assay

The migratory potential of U251 cells in the presence or absence of gedunin was assessed using a scratch assay.^32^ U251 cells were seeded (2 × 10^5^ cells/well) in a 6‐well plate. A line was made in the cell monolayer using a 200‐μL pipette tip. The plates were washed twice with PBS. The glioma cells were subjected to gedunin treatment for 48 h and imaged with a Motic microscope AE2000 (PA, USA).

### 2.9 Measurement of LDH release

We employed the release of LDH as a measure of cytotoxicity. The experiment was carried out in glioma cells treated with gedunin for 72 h. The amount of LDH released by the cells indicates cellular injury and compromised cells. The LDH released was quantitated at 490 nm using a spectrophotometer (Promega Glomax, Madison, WI). The percentage of LDH released was calculated. The experiments were conducted three times, and the means were used to calculate the amount of LDH released.^33^

### 2.10 Statistical analysis

All experimental studies were carried out in three biological replicates. GraphPad Prism 9 (La Jolla, CA, USA) was used for statistical analyses, and the data were given as means ± SEM. A comparison of the means was done using one‐way ANOVA and Dunnett's test.

## 3 RESULTS

We analyzed the effects of gedunin on glioblastoma cell lines and elucidated the possible mechanisms responsible for its biological effects. The results obtained from the experiments probing the biological cellular actions of gedunin on various glioblastoma cell lines are discussed below.

### 3.1 Gedunin treatment inhibits glioma cell viability

**FIGURE 2:**
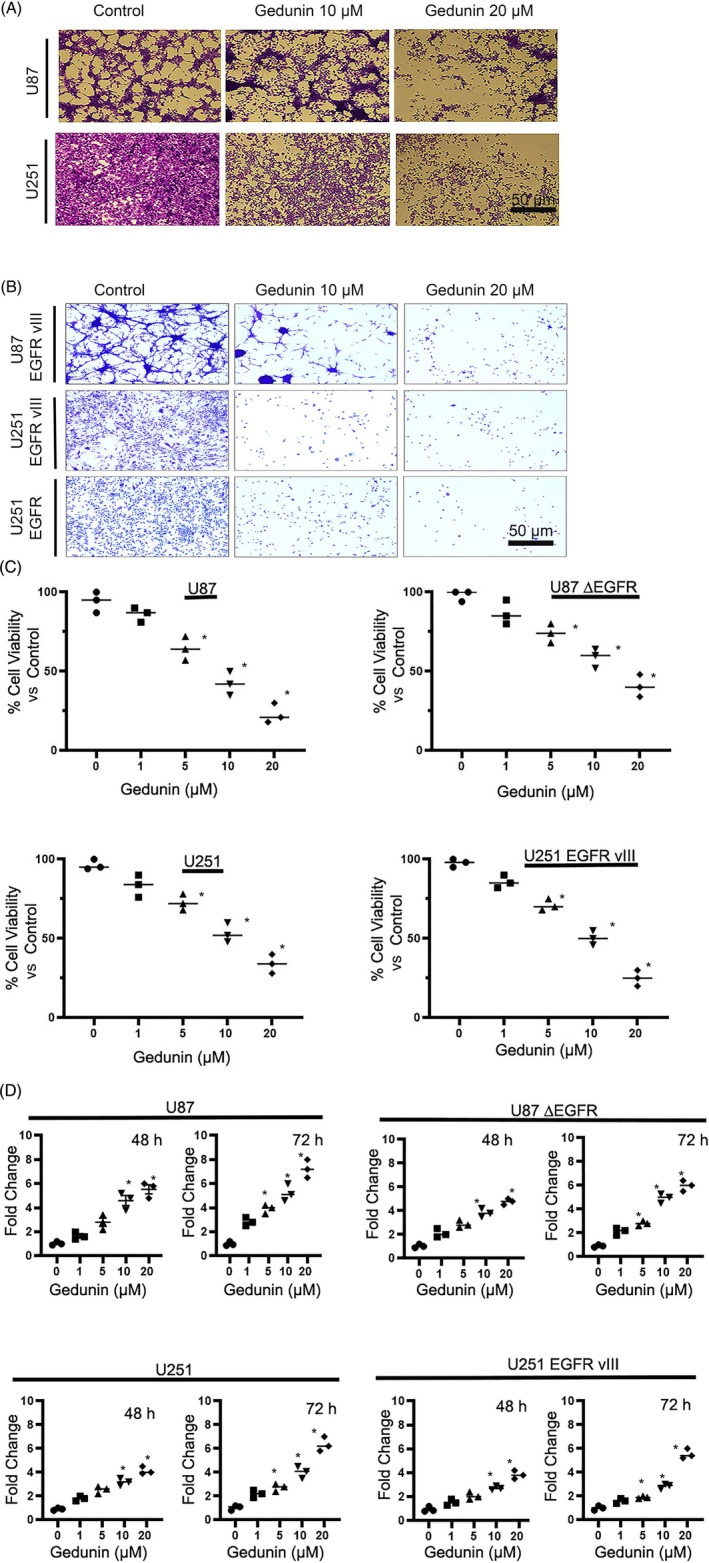
(A) Effects of gedunin (10–20 μM) on cell morphology and toxicity in U87 and U251 glioblastoma cells. Bar scale 50 μM. (B) The effects of gedunin (10–20 μM) on cell morphology and cytotoxicity in U87 EGFR vIII, U251 EGFR vIII, and U251 EGFR cells. Bar scale 50 μM. (C) The effects of gedunin (10–20 μM) on glioblastoma cell lines cell count using the trypan blue exclusion assay on CytoSmart. The treatment showed a significant (*p < .05) concentration‐dependent decrease in cell count in glioblastoma cell lines. The cells were treated with gedunin for 16 h. All data are represented as the mean ± SD of experiments performed in three biological replicates. (D) Cytotoxicity of gedunin (0–20 μM) in U87 and U251 cells, U87 EGFR vIII, and U251 EGFR vIII cells as measured by a lactate dehydrogenase (LDH) assay after 48 and 72 h of treatment. *Denotes significant (*p* < .05) difference in LDH release. All data are represented as the mean ± SD of experiments performed in three biological replicates.

The effect of gedunin (10 and 20 μM) was tested on the morphology of U87 and U251 glioblastoma cell lines (Figure 2A). Gedunin decreased cell viability and caused changes to the morphology of the U87 and U251 cells. Additionally, the effects of gedunin on glioma cells overexpressing EGFR and EGFR vIII were probed. We observed that gedunin did not significantly alter the morphology of Normal Human Astrocytes (NHA; see Data S1A) but remarkably modified the morphology of glioblastoma cells (Figure 2B). We further examined how gedunin affected the growth and induced cytotoxicity in these cells via a trypan blue exclusion assay. In the trypan blue assay, cells with uncompromised cell membranes exclude trypan blue, while compromised cells are penetrated by trypan blue. Our data showed that with increasing concentrations of gedunin, the cells lack the ability to exclude trypan blue and thus show a concentration‐dependent positivity for trypan blue (Figure 2C).

The release of substantial levels of LDH represents weakened or damaged cells. The cytotoxic effects of gedunin on LDH release were quantitated using an LDH assay kit. Gedunin (0–20 μM) induced cytotoxicity in parental and glioma cells overexpressing EGFR and EGFR vIII at 48 and 72 h (Figure 2D).

### 3.2 Gedunin abrogates cell viability and migration

**FIGURE 3:**
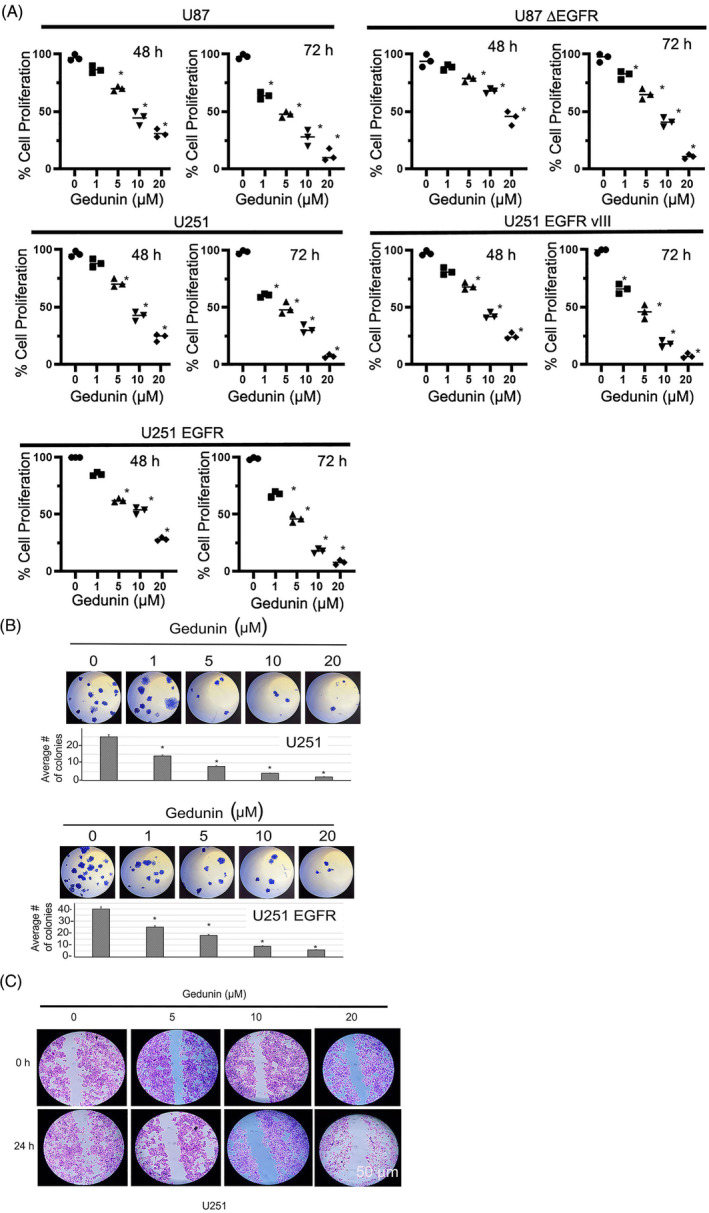
The effects of gedunin (1–20 μM) on the proliferation of U87 MG, U87 EGFR vIII, U251, U251 EGFR vIII, and U251 EGFR cells as measured by MTT assay after 48–72 h of treatment. The data is represented as the mean ± SEM. *Denotes significance (*p* < .05) in proliferation. Experiments were performed in three biological replicates. (B) The effects of gedunin (1–20 μM) on colony assay in U251 glioblastoma cell line. There is a concentration‐dependent decrease in colonies formed with treatment compared to control untreated cells. *Denotes significant (*p* < .05) difference in colonies of treated cells compared to untreated control cells. (C) The effects of gedunin (5–20 μM) on wound healing assay in U251 glioblastoma cell lines. Gedunin decreases the ability to migrate and close the wound. Bar scale 50 μm. Experiments were performed in three biological replicates.

Glioblastoma is characterized by the propensity of increased cell proliferation and its ability to invasively infiltrate the normal brain. The MTT assay was used to ascertain the effects of gedunin on the cellular proliferation of glioblastoma cells (U87 and U251). This assay assesses the propensity of cells to produce formazan from MTT. This conversion is a measure of cellular proliferation. We observed a decrease in the proliferation of gedunin‐treated (0–20 μM) cells. Additionally, the EGFR is one of the client proteins for HSP90. We probed the effects of gedunin, an HSP90 inhibitor, on glioma cells overexpressing EGFR or the truncated EGFR (EGFR vIII). Our results show that treatment with gedunin resulted in a reduction in cell proliferation (Figure 3A).

To further test the antiproliferative potential of gedunin in glioma cells, we carried out a colony formation assay in U251 and U251 EGFR cells. Gedunin (1–20 μM) concentration‐dependently decreased the number of colonies formed (Figure 3B). Glioblastoma cells tend to invade normal brain tissues; therefore, we tested the anti‐migratory effects of gedunin in glioma cell line U251 using a scratch assay. A straight line was created in monolayer cells, followed by treatment with gedunin for 48 h. The control experiment showed that the cells were migrating through the wound created. On the other hand, cells treated with gedunin inhibited cell migration. Concentrations of 10 and 20 μM inhibited (*p* < .05) the migration of the cancer cells (Figure 3C).

### 3.3 Gedunin modulates key survival signaling proteins

**FIGURE 4:**
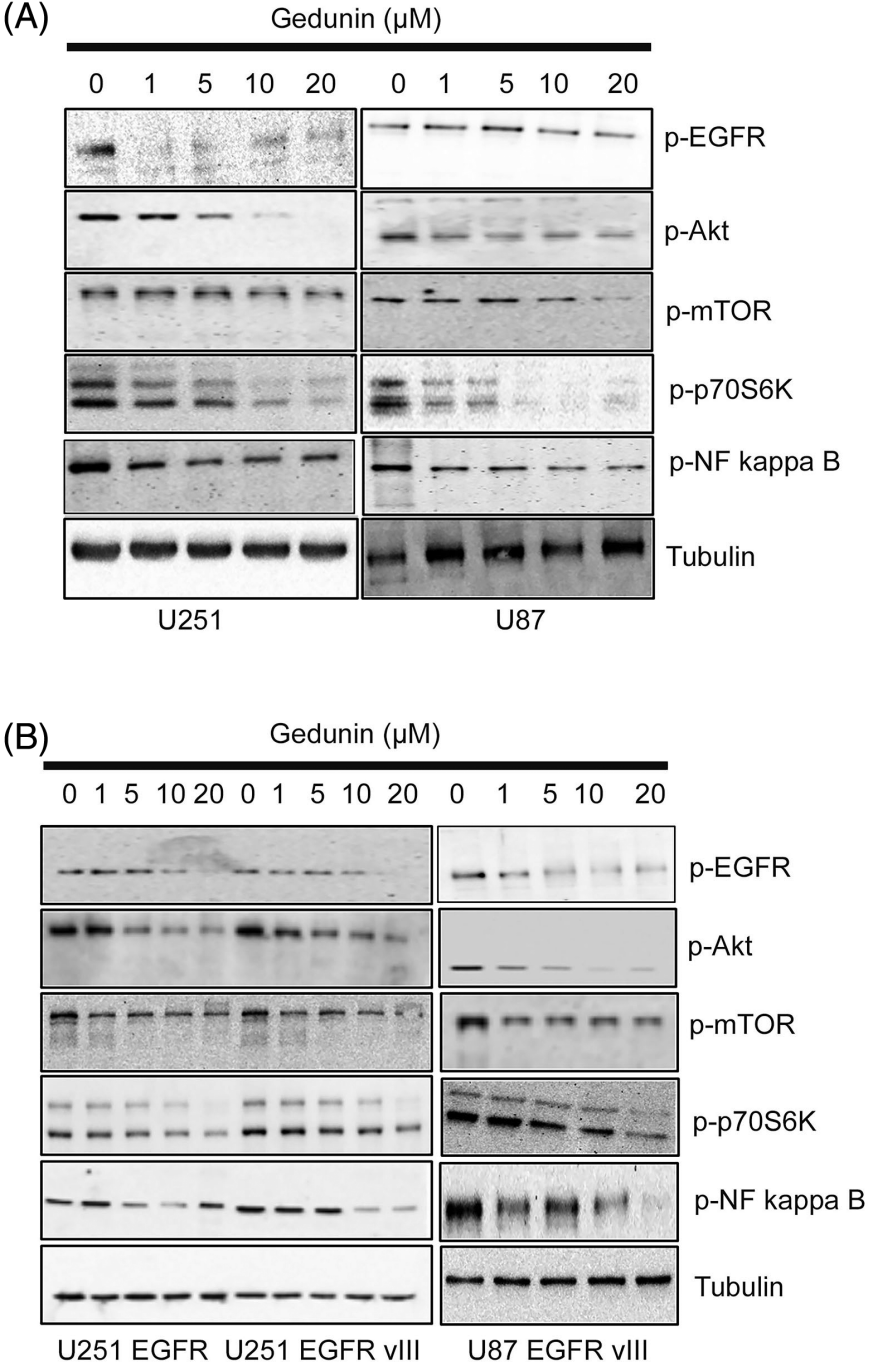
The effects of gedunin (0–20 μM) on the protein expression profile of phospho EGFR, Akt, NF kappa B, and p‐mTOR in U87 and U251 glioblastoma cell lines. The cells were treated with gedunin for 16 h. These figures are representative of Western blot images of three biological replicates. (B) The effects of gedunin (0–20 μM) on the protein expression profile of phospho EGFR, Akt, NF kappa B, and p‐mTOR in U87 EGFR vIII, U251 EGFR vIII, and U87 EGFR vIII glioblastoma cell lines. The cells were treated with gedunin for 16 h. These figures are representative of Western blot images of three biological replicates.

HSP90 is known to regulate EGFR and Akt.^11^, ^34^ The HSP90 inhibitor activity of gedunin is widely documented in the literature.^15^, ^18^, ^21^ Here, we explore the potential effects of gedunin on some key cellular signaling proteins. To test the impact of gedunin on HSP90 client proteins mediated signaling, glioblastoma cells (U87 MG, U251 MG, U251 EGFR, U251 EGFR vIII, and U87 EGFR vIII) were treated with gedunin (0–20 μM). Western blot analyses were carried out to probe the expression levels of the client proteins. We observed that treatment with gedunin downregulated the protein expression levels of p‐EGFR, p‐Akt, and p‐NF kappa B in both the parental cell lines and cells overexpressing the EGFR and EGFR vIII. The downregulation of these proliferative signaling proteins was concentration‐dependent. Overall, there was significant sensitivity to treatment with gedunin in all the cell lines (Figure 4A,B).

### 3.4 Gedunin induces cellular apoptosis in glioma cell lines

**FIGURE 5:**
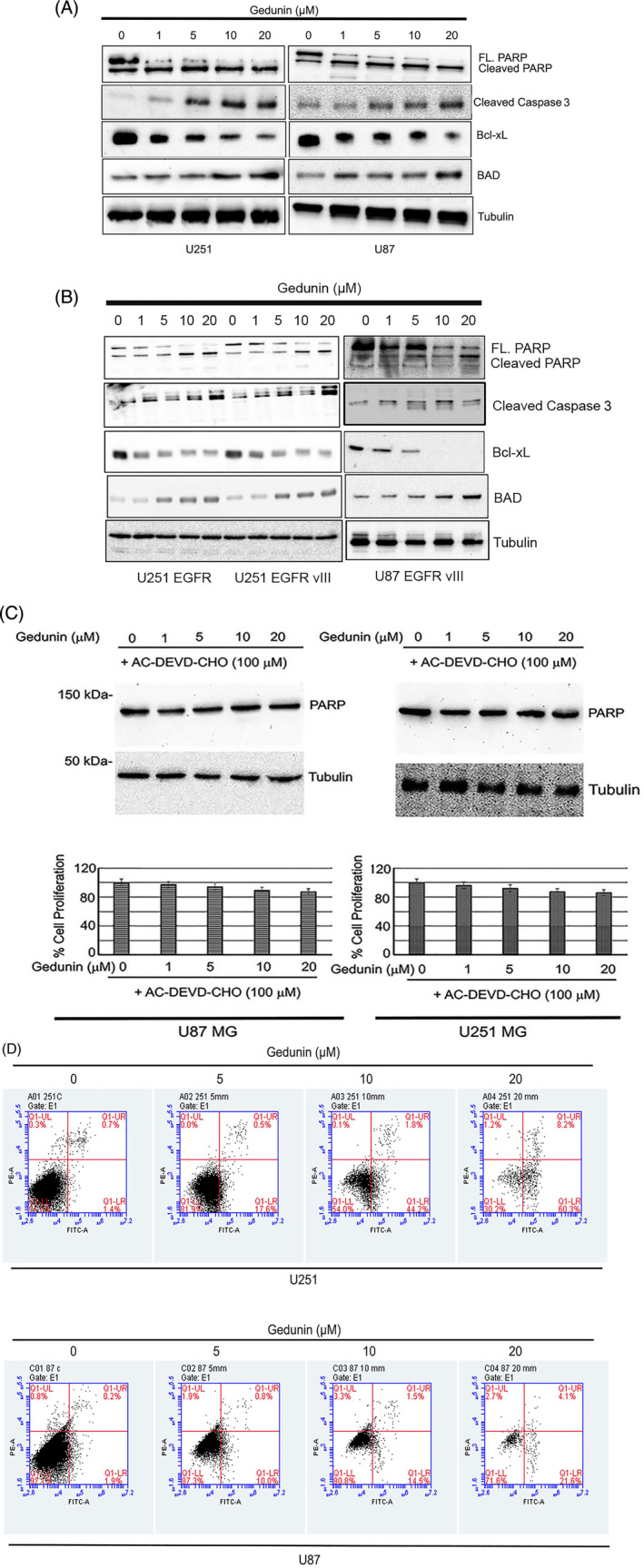
(A) The effects of gedunin (1–20 μM) on the expression profile of apoptotic protein expression profiles such as PARP, cleaved caspase 3, Bcl‐xL, and BAD in U87 and U251. The cells were treated with gedunin for 72 h. These figures are representative images of three biological replicates. (B) The effects of gedunin (1–20 μM) on apoptotic protein expression profiles such as PARP, cleaved caspase 3, Bcl‐xL, BAD in U87 EGFR vIII, U251 EGFR vIII, and U251 EGFR. The cells were treated with gedunin for 72 h. These figures are representative images of three biological replicates. (C) Caspase 3 inhibitor Ac‐DEVD‐CHO attenuates the effects of gedunin (0–20 μM) on the expression of cleavage of PARP in U251 MG and U87 MG cells. Caspase 3 inhibitor Ac‐DEVD‐CHO abrogates the decrease in cell viability induced by gedunin (0–20 μM) in U251 MG and U87 MG cells. These figures are representative images of three biological replicates. These results are not statistically significant. (D) Effects of gedunin on cell apoptosis in U251 MG and U87 MG cells. Gedunin (5–20 μM) induced significant (*p* < .05) apoptotic events using the BD Accuri C6 plus. These figures are representative images of three biological replicates.

In these experiments, we tested the potential of gedunin to promote cell death in glioblastoma cells. PARP, caspase 3 cleavage, and Bcl‐xL protein reduction are crucial markers of apoptotic signaling activation. Their activation is an essential indicator of programmed cell death. We examined the ability of gedunin to induce cell death in glioblastoma cell lines. To do this, we explored how gedunin affects protein levels of both proapoptotic and antiapoptotic proteins such as BAD, Bcl‐xL, cleaved caspase 3, and cleaved PARP by Western blot analyses. We initiated treatment with increasing concentrations of gedunin (0–20 μM) in both parental cell lines and cell lines overexpressing the EGFR and the EGFR vIII. Our data revealed that in all the cell lines tested, there was an increase in PARP cleavage at concentrations of 10–20 μM of gedunin. Similarly, we also observed an increase in caspase 3 cleavage in all the cell lines. Furthermore, we observed that treatment with gedunin increased the expression level of BAD and downregulated Bcl‐xL. These data suggest that gedunin induces PARP cleavage (Figure 5A,B), leading to cell death. Additionally, we determined the Bax to Bcl‐2 ratio. Our result showed an increase in this ratio (see Data S1B). The increase in this ratio indicates an induction of apoptosis.^35^, ^36^

To further examine the contribution of caspases in gedunin‐induced programmed cell death, we incubated glioma cell lines treated with gedunin and the caspase 3 inhibitor, Ac‐DEVD‐CHO. Our data showed that pretreatment with Ac‐DEVD‐CHO prevented gedunin from inducing cell death as measured by PARP cleavage (Figure 5C upper panel). Gedunin treatment in the presence of the caspase 3 inhibitor did not significantly decrease cellular growth (Figure 5C lower panel).

### 3.5 Gedunin induces apoptotic events in glioma cells

FITC‐PI stains were used to assess the extent of apoptosis in the cells. Our results showed that gedunin (5–20 μM) caused a robust increase in both early and late apoptotic events compared with control in both cell lines tested. In the U251 cell line, we observed that gedunin at doses of 5–20 μM caused early apoptosis (17.6%, 44%, and 60%, respectively) compared with the control. We observed a similar trend in the U87 cell line treated with gedunin (5–20 μM), producing 10%, 14%, and 22%, respectively, compared with the control (Figure 5D). We also observed that gedunin (5–20 μM) induced a concentration‐dependent increase in late apoptotic events of 0.5%, 1.8%, and 8.2%, respectively, compared with the control in U251. We observed a similar trend in the U87 cell line treated with gedunin (5–20 μM), producing 0.8%, 1.5%, and 4.1%, respectively, compared with the control (Figure 5D).

## 4 DISCUSSION

Here, we explored the biological effects and the mechanistic pathways of the impact of gedunin on growth‐mediated cellular signaling proteins. Our study demonstrates that gedunin decreased cell proliferation and viability in the parental and glioma cell lines overexpressing EGFR and the truncated EGFR vIII. The effects were more robust at concentrations of 10 and 20 μM. These findings were similar to the reported effects of gedunin on ovarian, breast, and pancreatic cancers.^15^, ^22^, ^37^ Gedunin has been established as an HSP90 inhibitor and modulates the role of the EGFR, Akt, Raf, MEK, and FAK, PDGFR, Cdk‐4, ‐6, and ‐9 signaling proteins.^11^, ^34^ To determine the essential growth‐mediated cellular proteins facilitating the decrease in cellular proliferation, we carried out Western blot analyses on the parental cells and cells overexpressing the EGFR and EGFR vIII. We observed that treatment with gedunin (1–20 μM) downregulated the expression of the phosphorylated levels of EGFR, Akt, and NF kappa B. These proteins are key regulators of cell growth. Downregulating these proteins could be essential in the observed biological response to gedunin. The EGFR is overexpressed and amplified in GBM and serves as a driver for increased growth and proliferation^38^, ^39^; thus, attenuating its phosphorylated form can decrease cell proliferation and migration.

Studies have suggested that nuclear factor kappa‐light‐chain‐enhancer of activated B cells (NF‐κB) is expressed in glioblastoma. The increased expression level of NF‐κB correlates with malignancy, resulting in a poor outcome in GBM patients.^40^ Similarly, Akt signaling is involved in proliferation, cell death, and cancer growth.^41^ Gedunin downregulates Akt phosphorylation. This downregulation could account for the observed decrease in cellular growth of these cancer cell lines. Our results show that NF‐κB phosphorylation is attenuated following treatment with gedunin. Taken together, the attenuation of these critical signaling proteins could mediate the effects of gedunin on glioblastomas.

Apoptosis is an intrinsic programmed cell death that occurs in several pathological conditions. We further explored the apoptotic effects of gedunin using cell cycle analysis. Our data showed that increasing the concentration of gedunin progressively resulted in greater percentages of cells in early apoptosis. Gedunin, at the concentrations tested, induced apoptosis in the glioblastoma cell lines. Treatment with gedunin modifies the expression levels of Bcl‐xL protein. The Bcl‐xL protein is antiapoptotic and thus inhibits cellular death.^42^ Gedunin decreased Bcl‐xL in the tested glioma cell lines, suggesting this compound promotes cell death via diminished expression of the Bcl‐xL protein. Bax to Bcl‐2 ratio expression represents cell death alterations, which regulates cell response to an apoptotic injury; an increased Bax/Bcl‐2 ratio causes the loss of cellular resistance to apoptotic activation, which leads to increased programmed cell death and attenuation of tumors (see supplemental data 1B).^43^, ^44^, ^45^, ^46^, ^47^

A critical requirement for the use of chemotherapeutic agents in the management of glioblastoma is the propensity of the drug to cross the blood–brain barrier. Based on its reported pharmacokinetic profile, gedunin is poorly absorbed orally with limited CNS penetration.^24^, ^25^ This agent would have to be formulated as a nanomedicine to enhance its potential for use in glioblastoma.

Our data demonstrate the potential of gedunin to initiate the activation of effector caspases, leading to the inhibition of the DNA repair mechanism. We also observed that gedunin induced PARP and caspase 3 cleavage. Both PARP and caspase 3 are the major markers of apoptosis.^48^ These cleavage events are characteristic of cell death.^49^ Furthermore, our findings show that gedunin‐treated GBM cells had an increase in both early and late apoptotic events, which is indicative of the cells undergoing apoptosis.

## 5 CONCLUSION

Gedunin attenuated glioblastoma cell proliferation and induced cellular apoptosis. We observed the decrease in phosphorylated EGFR, Akt, and NF Kappa B as key signaling intermediates that are perturbed following treatments with varying concentrations of gedunin. Furthermore, we observed that increasing concentrations of gedunin resulted in PARP and caspase 3 cleavage leading to apoptosis. Additionally, this study has not been conducted in vivo in small animals. Additionally, gedunin, like many other phytochemicals, does not appreciably cross the blood–brain barrier, so we are working with our collaborators to develop nanoparticles of gedunin and test its ability to cross the blood–brain barrier.

## AUTHOR CONTRIBUTIONS

**Samson Amos:** Conceptualization; investigation; methodology; validation; writing – review and editing; formal analysis; project administration; supervision. **Michael Stouffer:** Methodology; formal analysis; writing – original draft; investigation. **Elizabeth Wandling:** Investigation; methodology; writing – original draft; formal analysis. **Lindsay Dickson:** Investigation; writing – original draft; methodology; formal analysis. **Stacy Lin:** Investigation; writing – original draft; methodology; formal analysis. **Huanyun Duan:** Investigation; writing – original draft; methodology; formal analysis. **Erika Powe:** Investigation; writing – original draft; methodology; formal analysis. **Denise Jean‐Louis:** Conceptualization; investigation; methodology; validation; writing – review and editing; formal analysis; supervision. **Amit K. Tiwari:** Conceptualization; investigation; writing – review and editing; methodology; validation; formal analysis; supervision.

## FUNDING INFORMATION

The grant for this study was provided by Cedarville University School of Pharmacy (CUSOP‐2019‐20‐04) to Dr. Samson Amos.

## CONFLICT OF INTEREST STATEMENT

The authors have no conflicts to declare.

## ETHICS STATEMENT

This study was conducted in line with accepted biosafety procedures.

## Supporting information


**Data S1.** A: Effects of gedunin (10–20 μM) on the morphology of Normal Human Astrocytes (NHA).
**Data S1.** B: Effects of gedunin on Bcl2/Bax expression and relative ratios in glioblastoma cell lines.

## Data Availability

The data that support the findings of this study are available from the corresponding author upon reasonable request.
