# Supplementary material for: Gedunin modulates cellular growth and apoptosis in glioblastoma cell lines
Source: Cancer Rep (Hoboken). 2024 May 4;7(5):e2051. doi: 10.1002/cnr2.2051 (PMC11069102; doi:10.1002/cnr2.2051)
Supplement: Supplementary file 1 — Data S1. A: Effects of gedunin (10–20 μM) on the morphology of Normal Human Astrocytes (NHA). Data S1. B: Effects of gedunin on Bcl2/Bax expression and relative ratios in glioblastoma cell lines. [file CNR2-7-e2051-s001.docx]

Supplementary Data 1

A: Effects of gedunin (10-20 µM) on the morphology of Normal Human Astrocytes (NHA)

B. Effects of gedunin on Bcl2/Bax expression ratio in glioblastoma cell lines.
